# Purification of native HBHA from *Mycobacterium avium* subsp*. paratuberculosis*

**DOI:** 10.1186/1756-0500-6-55

**Published:** 2013-02-07

**Authors:** Louise H Lefrancois, Christelle C Bodier, Sophie Lecher, Florence B Gilbert, Thierry Cochard, Grégoire Harichaux, Valérie Labas, Ana Paula Teixeira-Gomes, Dominique Raze, Camille Locht, Franck Biet

**Affiliations:** 1 INRA, UMR ISP 1282 Infectiologie et Santé Publique, Nouzilly F-37380, France; 2 CNRS UMR, Lille, 8204, France; 3 INSERM, Lille, U1019, France; 4 Institut Pasteur de Lille, Lille, France; 5 Univ Lille Nord de France, Lille, F-59000, France; 6 INRA, UMR85 Physiologie de la Reproduction et des Comportements Plate-forme d’Analyse Intégrative des Biomarqueurs, Nouzilly, France; 7 CNRS, UMR, Nouzilly, 7247, France; 8 IFCE, Nouzilly, F-37380, France

**Keywords:** *Mycobacterium avium* subsp. *paratuberculosis*, HBHA, heparin-Sepharose chromatography

## Abstract

**Background:**

Paratuberculosis remains today a major global problem in animal health, especially for dairy cattle. However, the diagnosis of its etiologic agent, *Mycobacterium avium* subsp. *paratuberculosis* (Map), still lacks sensitivity because of the lack of available antigens. Little is known about the virulence factors for this pathogen. In this study we have developed a method to produce and purify the heparin-binding hemagglutinin (HBHA), a major adhesin of *Mycobacteria*, from a culture of Map.

**Findings:**

For this extremely slow-growing *Mycobacterium*, a culture was established in a 3-liter bioreactor. Using the bioreactor the amount of the Map biomass was increased 5-fold compared to a classical culture in flasks. The map-HBHA was purified from a Map lysate by heparin-Sepharose chromatography on HiTrap columns. Binding of map-HBHA onto heparin-Sepharose can be reduced in the presence of salt. Consequently, all steps of sample preparation and column equilibration were carried out in 20 mM Tris–HCl (pH 7.2). The map-HBHA was eluted by a linear NaCl gradient. High resolution mass spectrometry analyses revealed that the native form of map-HBHA has posttranslational modifications, including the removal of the initiation methionine, acetylation of the alanine residue at the N-terminal extremity and the presence of methylated lysines in the C-terminal domain of the protein.

**Conclusions:**

An optimized culture of Map in a bioreactor was established to purify the native map-HBHA from a Map lysate by heparin-Sepharose chromatography. The availability of this antigen offers the possibility to study the structure of the protein and to examine its role in pathogenicity, in particular to better understand the specific interactions of Map with the intestinal tissue. The map-HBHA obtained in its native immunogenic form may also be useful to improve the diagnostic test, especially for the development of a new T-cell-based interferon gamma release assays.

## Findings

### Background

*Mycobacterium avium* subsp. *paratuberculosis* (Map) is the etiologic agent of a severe granulomatous inflammatory bowel disease in ruminants, known as Johne’s disease or paratuberculosis [1,2]. This enzootie remains today a major animal health problem with a high global prevalence causing significant economic losses in the cattle industry [2-6]. One characteristic feature of Map is its adaptation to the gastrointestinal tract of ruminants. However, the molecular and cellular mechanisms of its interactions with M cells [7] and epithelial cells [8] are not yet fully elucidated. *Mycobacterium*-epithelium interactions, much better studied with *Mycobacterium tuberculosis*, involve an adhesin called heparin-binding hemagglutinin (HBHA), which plays a crucial role in the binding of the mycobacteria to epithelial cells and other non-phagocytic cells [9,10]. HBHA is an adhesin that binds sulphated carbohydrates, such as heparin and heparan sulfate, present on the surface of various eukaryotic cells types, via its C-terminal domain containing lysine-rich motifs [11]. Native HBHA from *M. tuberculosis* carries methyl groups added post-translationally, which are important for its antigenicity [12]. Several investigations have demonstrated that a HBHA-specific IFN- γ response, indicative of latent *M. tuberculosis* infection and protective immunogenicity depends on the methylation of the antigen [13-16].

Comparative genomics suggests that HBHA is also present in Map. In order to study the biochemical characteristics of the HBHA from Map (map-HBHA) and to evaluate its potential in diagnostics and vaccine development, it is essential to have access to native map-HBHA. Our first attempts to purify map-HBHA by heparin-Sepharose chromatography under conditions used to purify HBHA from *M. tuberculosis* failed to isolate the map-HBHA. In addition, the purification was hampered by the difficulty to obtain sufficient biomass of Map due to the extremely slow growth of this microorganism.

In this study we established a new method of Map culture, using a bioreactor, which substantially increased the biomass yield and shortened the time of culture. In addition, we describe a new purification procedure, which led to the isolation of native map-HBHA.

### Results and discussion

#### Optimization of Map biomass production in a bioreactor

**Figure 1:**
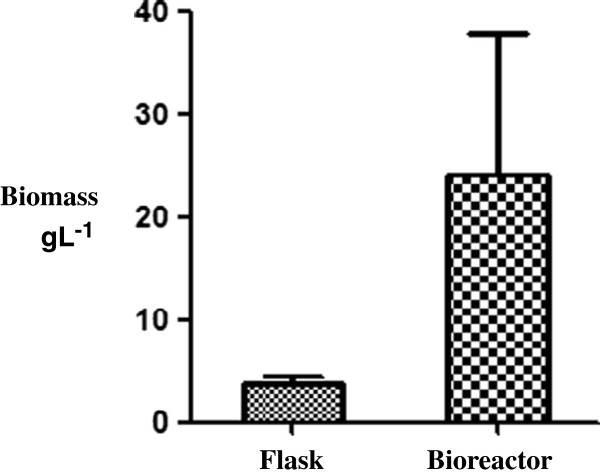
**Increasing biomass production using a bioreactor. **Cultures of Map K-10 (ATCC BAA-968) in flask or bioreactor were inoculated with a pre-culture at an OD_600 nm _of 0.06. Both cultures were maintained at 37°C during 40 days under agitation and air in the bioreactor and under static conditions in the flask. The bacteria were harvested by centrifugation and the biomasses were weighed. The figure represents the average of three independent experiments (+/− standard deviations).

Map is one of the slowest-growing *Mycobacteria* with a mean generation time of 1.38 days [17]. Map cultures thus require long time periods with limited opportunities for recording and correcting parameters. Furthermore, the final product of these cultures was shown to contain high proportions of dead bacilli that may reduce the quality of the proteins harvested [18]. To circumvent these difficulties, we chose to investigate the growth of Map in a bioreactor to increase the biomass, standardize the culture conditions and control potential contaminations. Previous studies using bioreactors have described the standardization of *M. bovis* BCG cultures for vaccine production [19,20]. Growth of Map in a bioreactor was compared to conventional culture flasks inoculated in parallel with the same Map pre-culture at an OD_600nm_ of 0.06. After 40 days, the cultures were centrifuged and the harvested biomasses were weighed. As shown in Figure 1, the biomass harvested from the bioreactor was significantly more important in comparison to the conventional culture in flasks. Using the bioreactor we obtained, as an average of three independent experiments, five times more bacteria than with flask cultures (4 versus 20 g L^-1^).

#### Protein purification

**Figure 2:**
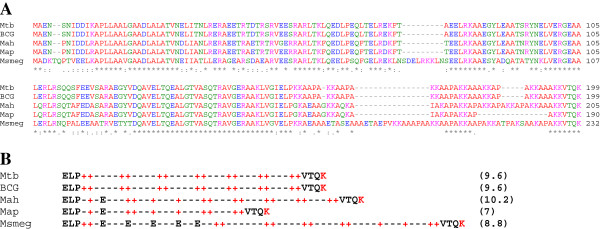
**Sequence alignment of different HBHA proteins. **(**A**) Multiple sequence alignment was performed using the Clustal W program with the BLOSUM64 matrice allowing gaps (−). * indicate identical residues. : indicate conserved substitutions. ; indicate semi-conserved substitutions. Mtb, *M. tuberculosis*; BCG, *M. bovis* BCG; Mah*, M. avium* subsp*. hominissuis* and Map*, M. avium *subsp*. paratuberculosis*; Msmeg, *M smegmatis*. (**B**) Distribution of charges within the C-terminal domain of HBHA. The isoelectric point of each protein is indicated in brackets. Color code: Polar charged residues HKR (Pink); Polar residues GNQSTY (Green); Polar negative residues DE (Blue) and other residues (Red).

Our first attempts to purify map-HBHA by heparin-Sepharose chromatography under conditions used to purify HBHA from *M. tuberculosis* have been unsuccessful. This may be due, at least partly, to the nature of the protein. Although the alignment of the HBHA amino acid sequences from different mycobacterial species indicates that these molecules are highly conserved, the C-terminal lysine-rich domain is rather divergent (Figure 2A). These differences may modify the affinity of the proteins for the heparin-coupled matrix, due to the differences in the distribution of the positively charged lysines involved in the cation- and anion-exchange chromatography (Figure 2B). In a previous study, we have shown that the isolation of the *M. smegmatis* HBHA requires a modification of the elution parameters, as its C-terminal end is less positively charged, compared to that of the *M. tuberculosis* HBHA [21]. As indicated in Figure 2B, the C-terminal sequence of the map-HBHA contains one acidic residue and 9 positively charged lysines compared to 14 positively charged lysines in HBHA from *M. tuberculosis*. The predicted isoelectric point (7) is lower than that calculated for HBHA of *M. tuberculosis* (9.6) or *M. smegmatis* (8.8), suggesting that the binding and elution parameters have to be adjusted. Because of the slow growth of Map, the chromatography conditions were first established with a recombinant form of map-HBHA producing in *E. coli*.

#### Heparin-sepharose column

**Figure 3:**
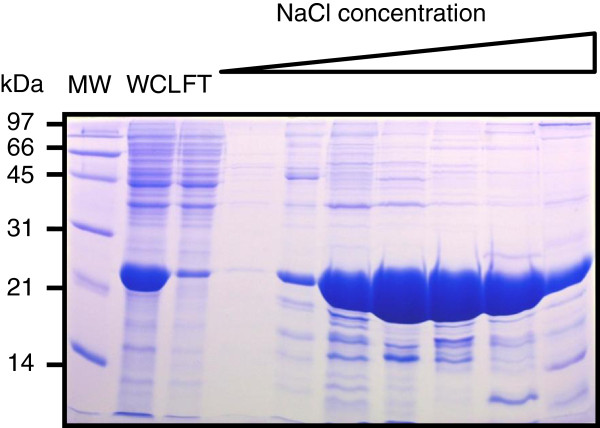
**Heparin-Sepharose chromatography of recombinant map-HBHA produced in *E. coli.*** A culture of recombinant *E. coli *producing map-HBHA was sonicated in 0.5 × PBS. The soluble material was loaded onto a heparin-Sepharose HiTrap column and eluted using a 0-1 M NaCl gradient. Whole cell lysate (WCL), flow-through material (FT) and eluted material were analyzed by SDS-PAGE. The fractions eluted between 100 and 350 mM NaCl contained map-HBHA as determined by HR-MS/MS. The molecular weights (MW) expressed in kDa are indicated in the left margin.

First, a lysate of recombinant *E. coli* producing map-HBHA was applied onto two types of heparin-Sepharose columns in 0.5 × PBS: the prepacked HiTrap column or heparin-Sepharose CL6B poured into a Pharmacia column. As shown in Figure 3, the recombinant map-HBHA was successfully purified from a lysate of *E. coli.* It was eluted from the heparin-Sepharose column at low salt concentrations, starting from 100 mM NaCl. Although both columns showed similar performances with a lysate containing large amounts of recombinant map-HBHA, the HiTrap column, more reproducible in our hands, was preferred.

#### Buffer conditions

**Figure 4:**
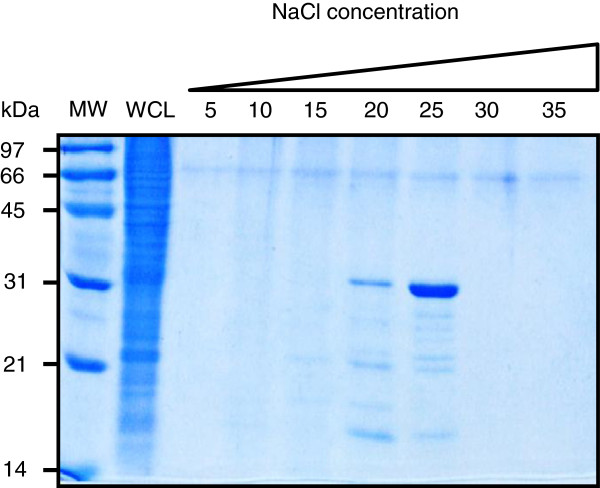
**Heparin-Sepharose chromatography of a Map lysate in PBS buffer. **A Map K-10 culture obtained using the bioreactor was centrifuged and the pellet was resuspended in 0.5 × PBS. The bacteria were then sonicated and centrifuged. The whole cell lysate (WCL) was applied onto a heparin-Sepharose HiTrap column and eluted using a 0-1 M NaCl gradient. WCL and the indicated elution fractions were analyzed by SDS-PAGE. Fractions 20 to 25, eluted around 500 mM NaCl, contained map-Lbp/Hlp, as determined HR-MS/MS. The molecular weights (MW) expressed in kDa are indicated in the left margin.

**Figure 5:**
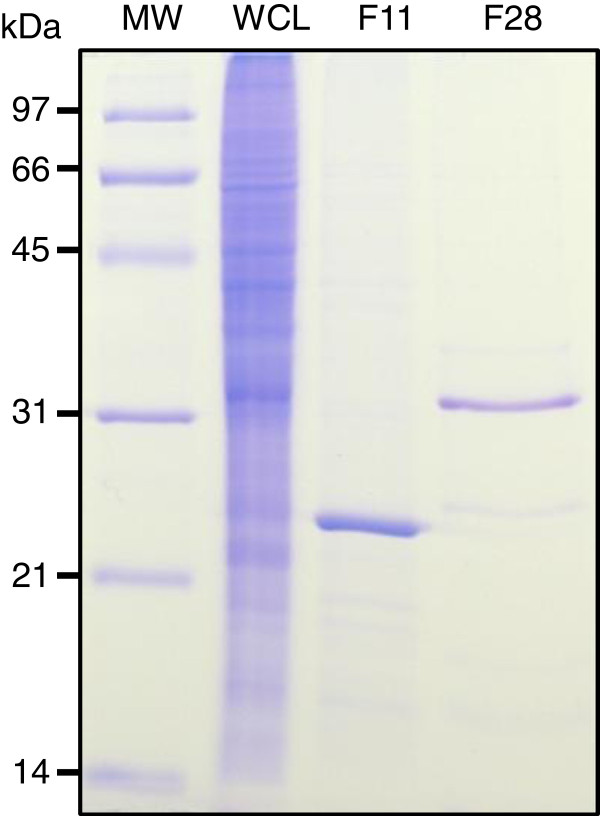
**Native map-HBHA purified by heparin-Sepharose chromatography in Tris–HCl buffer. **Using a 20 mM Tris-HCl (pH 7.2) buffer a whole cell lysate (WCL) of Map was subjected to heparin-Sepharose HiTrap chromatography. SDS-PAGE analysis showed that the material eluted in fraction 11 at ca. 200 mM NaCl contains map-HBHA, as determined by HR-MS/MS. The map-Lbp/Hlp was identified in fraction 28, eluted at ca. 500 mM NaCl. The molecular weights (MW) expressed in kDa are indicated in the left margin.

Surprisingly, when the supernatant of a whole-cell lysate of Map was subjected to purification under the conditions established for recombinant map-HBHA, the native map-HBHA was not isolated. As shown in Figure 4 in late elution fractions only one protein corresponding to Lbp/Hlp, previously described to bind heparin with strong affinity [22], was eluted at 500 mM NaCl in this study. These results suggest that binding and elution parameters need to be changed to isolate native map-HBHA. The 0.5 × PBS loading buffer was thus replaced by a 20 mM Tris–HCl (pH 7.2) buffer to reduce the presence of salt that can inhibit the binding of the protein to the matrix. The map-HBHA prepared in 20 mM Tris–HCl (pH 7.2) was able to efficiently bind the heparin-Sepharose matrix and was eluted at approximately 200 mM NaCl (Figure 5). The early elution of map-HBHA in the NaCl gradient suggests a relationship between the number of positive charges in the protein and the binding force to the sulfated sugars. As indicated in Figure 5, Lbp/Hlp eluted later, at 500 mM NaCl.

#### Mass spectrometry analysis

To ascertain the identities of the proteins eluted from heparin-Sepharose, High Resolution tandem Mass Spectrometry (HR-MS/MS) analyses using a bottom-up approach GeLC-MS/MS were carried out. These analyses confirmed that the 25-kDa protein eluted at 200 mM NaCl corresponds to map-HBHA and that the 31-kDa protein eluted at 500 mM NaCl corresponds to map-Lbp/Hlp. In addition, HR-MS/MS also revealed important characteristics of the native proteins purified from the Map culture (Table 1). Similar to the *M. tuberculosis* HBHA [23], native map-HBHA contains no initiation methionine at its N-terminus and that the first amino acid is thus an alanine residue. The cleavage of the initiation methionine is a post-translational common process in *Mycobacteria*[24,25]. The HR-MS/MS analyses also indicated that the amino-terminal alanine is acetylated. This post-translational modification already reported for other mycobacterial proteins may be an important regulator of protein functions [26,27].

**Table 1:** Identification of map-HBHA by mass spectrometry analysis

**Peptides identified by HR-MS/MS**	**Peptide position start - end**	**Peptide modification**
^2^A_ac_ENPNIDDLR^11^	2-11	Acetylated
^60^FQEDLPEQFTELR^72^	60-72	
^73^DKFTTEELR^81^	73-81	
^82^KAAEGYLEAATNR^94^	82-94	
^83^AAEGYLEAATNR^94^	83-94	
^109^LRSQTAFEDASAR^121^	109-121	
^111^SQTAFEDASAR^121^	111-121	
^111^SQTAFEDASARAEGYVDQAVELTQEALGTVASQTR ^145^	111-145	
^122^AEGYVDQAVELTQEALGTVASQTR^145^	122-145	
^151^AAK_me_LVGIELPGK^162^	151-162	Methylated
^154^LVGIELPGK^162^	154-162	
^154^LVGIELPGKAEAAGK_me_^168^	154-168	Methylated
^154^LVGIELPGKAEAAGKK^169^	154-169	
Peptides highlighted onto sequence^a^		

M*A*_*ac*_*ENPNIDDLR*APLLAALGAADLALATVNDLIANLRERAEETRAETRTRVEERRARLTK*FQEDLPEQFTELRDKFTTEELR*K*AAEGYLEAATNR*YNELVERGEAALQR*LRSQTAFEDASARAEGYVDQAVELTQEALGTVASQTR*AVGER*AAK*_*me*_*LVGIELPGKAEAAGK*_*me*_*K*AQKAIAKAPAKKAPAKRVTQK.

^a^The 13 peptides identified by HR-MS/MS, text in italic, correspond to 52% of sequence coverage.

**Figure 6:**
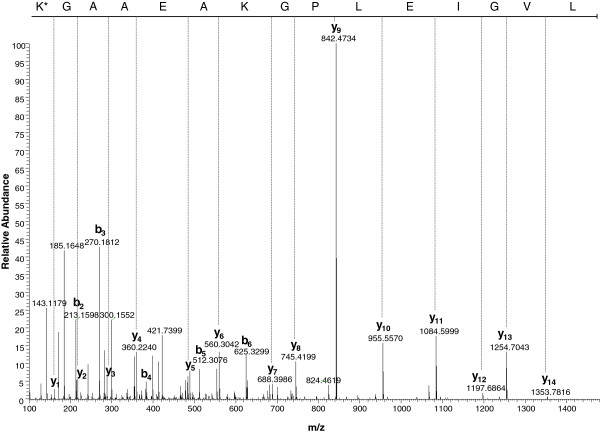
**Fragmentation spectrum of the HBHA**^**154**^**LVGIELPGKAEAAGK**^**168 **^**peptide with post-translational modification. **Shown is a spectrum obtained by nanoLC-MS/MS analysis with HCD fragmentation mode using LTQ Velos Orbitrap mass spectrometer. The precursor ion had an m/z 733.93 in charge state 2. The peptide sequence was obtained by all y ions (y_1 _to y_14_) and 5 consecutive b ions (b_2 _to b_6_). Moreover, the Δm = +14 Da on the K*168 indicated the presence of a methylation.

Another feature of native map-HBHA, revealed by HR-MS/MS, is the presence of methylated amino acids as indicated in Figure 6 and Table 1. Among the peptides analyzed in this study, the lysine residues 154 and 168 were mono-methylated. Other proteomic analyses are necessary to determine the complete methylation profile of the HBHA produced by Map. Although the exact role of the methylation remains to be elucidated, this post-translational modification was shown to be crucial for the immunogenicity of the *M. tuberculosis* HBHA. Only the native, methylated protein is able to induce strong IFN-γ secretion by peripheral blood mononuclear cells from latently infected human [14] and when administered in combination with strong Th1-promoting adjuvants only the methylated form was able to confer protection against *M. tuberculosis* in mouse models [16].

Altogether these data highlight the importance of having access to proteins in their native form with their specific post-translational modifications to understand their function, their location and to envisage their use as antigens for diagnostics or vaccine development.

### Conclusions

The objective of this study was to develop the conditions that allow us to purify native HBHA from a lysate of Map. To obtain sufficient biomass of Map, for which the culture is slow and tedious, a bioreactor culture method was developed. Under these conditions it was possible to obtain a biomass of 20 g per liter of culture, a 5-fold higher yield than in traditional culture conditions. Native map-HBHA was purified by heparin-Sepharose chromatography. HiTrap columns gave the best and most reproducible results. Due to the specific chemical characteristics of native map-HBHA, in particular the charges present in the C-terminal part of the protein, the Map lysate had to be prepared in 20 mM Tris–HCl (pH 7.2) to allow for optimal binding of map-HBHA to the heparin-Sepharose matrix. HR-MS/MS analyses revealed post-translational modifications, such as presence of an acetyl alanine residue at the N-terminus and methyl-lysines in the C-terminal part of the protein. In this study, we have thus optimized the Map culture conditions and the purification of native map-HBHA, which is therefore now available for structure-function studies and investigations for use in diagnosis or vaccine development against Map.

### Methods

#### Bacterial strains, growth conditions and DNA manipulations

*Mycobacterium avium* subsp. *paratuberculosis* (Map) strain K-10 (ATCC BAA-968) was grown at 37°C in Sauton medium supplemented with 2 mg L^-1^ of mycobactin J (IDVET, Montpellier, France). *E. coli* TOP10 (Invitrogen Carsbad, CA) and *E. coli* BL21(DE3) (Novagen, Darmstadt, DE) were grown in LB medium supplemented with 50 μg mL^-1^ kanamycin as appropriate. Restriction enzymes, T4 DNA ligase and other molecular biology reagents were purchased from New England Biolabs, Roche or Promega. PCRs were performed using a Bio-Rad thermal cycler model iCycler and the PCR products were sequenced by GenomExpress (Grenoble, France).

#### Cultivation of Map K-10 in bioreactor

A Map K-10 pre-culture was prepared in Sauton medium to obtain an initial optical density at 600 nm of 0.06. This was then inoculated either in 175 cm^2^-Greiner Bio-one Flasks maintained at 37°C in static conditions or in a 3-liter bioreactor (Setric Génie Industriel, Toulouse, France) constantly kept at 37°C with a stirring rate at 110 rpm and 12 L h^-1^ of air. During growth, serial time samplings were performed to determine absorbance at 600 nm. Once the stationary phase was reached, the bacteria were harvested by centrifugation at 7,000 × *g* for 15 min at 4°C and stored at −20°C until use.

#### Protein purification by heparin-Sepharose chromatography

A frozen Map pellet corresponding to 5 g was thawed, washed twice with 0.5 × PBS or 20 mM Tris–HCl (pH 7.2) and heated at 80°C during 30 min. Heat-killed bacteria were centrifuged at 10,000 × *g* for 20 min. The Map pellet was resuspended in 45 mL 0.5 × PBS or 20 mM Tris–HCl (pH 7.2) and sonicated intermittently in a Kimble HS N°45500-30 tube using a Branson Sonifier 250D for 20 min repeated 4 times, on ice. The soluble material obtained after centrifugation at 13,000 × *g* for 20 min at 4°C was filtered at 0.45 μ and applied onto the heparin-Sepharose matrix.

All chromatographic steps were carried out on the Biologic chromatography system (BioRad), at room temperature and the absorbance at 280 nm was continuously monitored during the purification. Two types of columns were investigated. One protocol used the HiTrap Heparin HP (1 mL, GE Healthcare) column (0.7 × 2.5 cm) prepacked with heparin-Sepharose. In the second protocol 1 g of heparin Sepharose CL-6B (GE Healthcare) resuspended in 5 mL of 0.5 × PBS or 20 mM Tris–HCl (pH 7.2) was poured into a 1.5 × 10 cm column (Pharmacia), according to the recommendations of the supplier. The columns were washed with 100 mL of 0.5 × PBS or 20 mM Tris–HCl (pH 7.2) until the absorbance at 280 nm was close to 0. The bound material was eluted by a 0-1 M NaCl linear gradient using a flow rate maintained at 0.6 mL min^-1^ and automatically collected in 1 mL fractions. Whole cell lysates, flow-through material and eluted fractions were analyzed by 12% sodium dodecyl sulfate-polyacrylamide gel electrophoresis (SDS-PAGE) performed according to Laemmli [28].

#### Cloning, sequencing and production of recombinant map*-*HBHA in *E. coli*

The map-HBHA-encoding gene (MAP3968) was amplified by PCR from chromosomal DNA using the *Taq* DNA polymerase (Promega) and two synthetic oligonucleotides (Sigma) with the following sequences: 5^′^- TATACATATGACCATGGCGGAAAACCCGAACATCG -3^′^ and 5^′^- ATATAAGCTTGGTACCCACGAGGTGGTTCACGCC -3^′^, containing *Nde*I and *Hin*dIII sites (underlined). The fragment was amplified after a short denaturation cycle of 3 min at 95°C by using 35 cycles as follows: 95°C for 30 s, 57°C for 30 s, and 72°C for 30 s with a final elongation cycle at 72°C for 10 min. The PCR product containing the map-HBHA-coding sequence was digested by *Nde*I and *Hin*dIII and then inserted into pET-24a(+) (Novagen), generating pET::map-HBHA. This plasmid was used to transform *E. coli* TOP 10 for sequencing and *E. coli* BL21(DE3) for protein expression. After transformation, *E. coli* BL21(DE3) cells were grown at 37°C in 250 mL LB broth supplemented with 50 μg mL^-1^ kanamycin. At an OD_600_ of 0.5, IPTG was added to a final concentration of 1 mM, and growth was continued for 4 h. The culture was then centrifuged at 7,000 × *g* for 15 min at 4°C. The pellet obtained was stored at −20°C until applied onto heparin Sepharose as described above.

The GenBank accession numbers for the map-HBHA (strains K-10 and ATCC19698) sequences are [mapK10_HBHA JX536266 and map19698_HBHA JX536267].

#### Identification of the purified Map proteins by high resolution mass spectrometry analysis

Proteins were digested in-gel with trypsin as previously described [29]. Peptides were analyzed by nanoLC-MS/MS using an Ultimate® 3000 RSLC coupled to a LTQ Velos Orbitrap Mass Spectrometer. Samples were loaded onto a trap column (Acclaim PepMap 100 C_18_, 100 μm inner diameter × 2 cm) at a flow rate of at 5 μL min^-1^ with 4% solvent B (0.1% formic acid, 15.9% water, 84% acetonitrile) and 96% solvent A (0.1% formic acid, 97.9% water, 2% acetonitrile). Peptides were fractionated using a nano-column (Acclaim PepMap C_18_, 75 μm inner diameter × 15 cm), with a nanoflow rate of 300 nl min^-1^ by applying a gradient of 4-55% B for 60 min. The LTQ Velos Orbitrap instrument was operated in a data-dependent mode with *R* = 30,000. In the scan range of m/z 300–1800, the 10 most intense peptide ions with charge states ≥2 were isolated for HCD fragmentation. Raw data files were converted to MGF. The data were searched against nr NCBI database (Eubacteria) using MASCOT search engine (version 2.3). Carbamidomethylcysteine was set as a fixed modification, and oxidation of methionine, N-terminal acetylation and methylation of lysine were set as variable modifications. Two missed cleavages were allowed. The tolerance of the ions was set to 10 ppm for parent and 0.1 Da for fragment ion matches. Mascot results were subjected to the Scaffold 3 software (version 3.2). Peptide identifications were accepted if they could be established at a greater than 95.0% probability as specified by the Peptide Prophet algorithm [30]. Protein identifications were accepted if they could be established at a greater than 99.0% probability and contained at least two identified peptides [31]. Identified post-translational modifications were validated using Scaffold PTM (version 2.0). Localization sites were accepted if they could be established at a greater than 99% probability as specified by the Ascore algorithm [32].

## Competing interests

The authors have no competing interests.

## Authors’ contributions

FB, LL and DR conceived of the study, participated in its design and coordination, collated and analyzed the data. FB, LL, DR and CL drafted the manuscript. CB, SL, TC, LL, FG, APTG, GH and VL participated in the laboratory and field work. All authors read, criticized and approved the final manuscript.
